# Protein Baggage: Toxicity of Organotin Tied to Proteasome Interference

**Published:** 2009-03

**Authors:** M. Nathaniel Mead

Organotins have been widely used as agricultural pesticides, anti-fungal agents, polyvinyl chloride stabilizers, industrial catalysts, and antifouling additives in boat paints. These tin-based chemicals, which have been detected in various environmental media, are lipophilic and thus capable of becoming increasingly concentrated as they pass up the food chain. A new study suggests that the toxic effects of organotins on living cells are mediated in part by inhibiting the function of the proteasome, a molecular structure that degrades unneeded or damaged proteins **[*EHP* 117:379–386; Shi et al.]**.

In eukaryotic organisms (animals, plants, fungi, algae, and plankton), more than 80% of intracellular proteins are degraded through the proteasome-mediated pathway. By interfering with proteasome function, organotins enable proteins to accumulate inappropriately. Because normal immune function and many cellular processes depend on the proteasome pathway, the organotin–proteasome interaction could help explain some of the adverse health effects of organotins—notably endocrine disruption, infertility, and immune dysfunction—that have been observed in wildlife and in animal studies. In addition, human exposure to organotins has been proposed as a possible risk factor for cancer (by inhibiting the cytotoxic function of natural killer cells), neurotoxicity, obesity, allergies, asthma, and altered reproductive development.

The researchers provide several lines of evidence suggesting that triphenyltin (TPT), a common organotin, binds to and blocks the activity of the proteasome by irreversibly inhibiting its protein-degrading activity. TPT was shown to have greater potency in this regard than seven other organotins examined by the authors. The investigators deduced that the tin present in TPT interacts with the N-terminal threonine of the proteasomal β5 subunit, possibly providing a specific target for organotins. Organotins have long been known to induce necrosis; the authors propose this may occur through caspase-dependent, DNA damage–independent cell death. In addition, the researchers assert that organotins most likely kill cells via a p53-independent pathway.

The new findings suggest that other previously identified potential targets of organotins, such as the transcription factor NFκB and the pro-apoptotic protein Bax, might be downstream of proteasome inhibition. The investigators further posit that inhibition of aromatase activity observed in organotin-exposed humans and animals—an effect linked to altered reproductive development—may be due to proteasome inhibition because such inhibition causes up-regulation of factors that suppress transcription of the *hCYP19*/aromatase gene.

